# Hematopoiesis: from start to immune reconstitution potential

**DOI:** 10.1186/s13287-015-0051-z

**Published:** 2015-04-11

**Authors:** Haydn C-Y Liang, Juan Carlos Zúñiga-Pflücker

**Affiliations:** Department of Immunology, University of Toronto, 1 King’s College Circle, Toronto, ON M5S 1A8 Canada; Sunnybrook Research Institute, 2075 Bayview Avenue, Toronto, ON M4N 3M5 Canada

## Abstract

The study of hematopoiesis has been a focus for developmental biologists for over 100 years. What started as a series of microscopic observations in different animal model systems has since evolved into studies of gene expression and regulation, and subsequent protein–protein interactions, cell surface protein expression profiling, and functional mapping of cell fates. In this review, we will discuss the milestone discoveries that have been achieved in the field of hematopoietic development, as well as the techniques that have been employed. Finally, we look toward the future and consider unresolved questions. We also reflect on one of the earliest realizations made in this area of study: that hematopoiesis is evolutionarily conserved, and as a consequence we reflect on the impacts of early and current discoveries and their clinical implications. The future direction of the study of hematopoietic stem cells will probably make use of pluripotent stem cells to yield specific immune cell lineages and eventual clinical applications.

## Introduction

The hematopoietic developmental process has been studied in detail since the early 1900s. While ER Clark first reported the observation of vascular generation in tadpoles in 1909, and Stockard described blood-vessel development and coined the term angioblast in fish embryos in 1915, it was not until 1920 that F Sabin described the formation of blood cells through the angioblast ‘liquefying’ within the aorta [1-3]. Since then several milestones have been achieved in understanding this complex and highly conserved developmental process.

## Basic hematopoietic development concepts

Mouse hematopoiesis was thought to develop initially in the yolk sac, where primitive hematopoietic development occurs [4]. This development is also often referred to as fetal hematopoiesis, and was demonstrated by the production of embryonic erythrocytes that still possess a nucleus, express fetal hemoglobin, and lack any lymphoid potential [5]. This initial wave of fetal hematopoietic development appears to have short-term reconstituting function, as demonstrated by Dieterlen-Lievre using chicken–quail chimeras [6]. The translatability of these experiments across different biological systems such as in *Xenopus laevis* and *Danio rerio* also demonstrates that this process is conserved across several species [2,7-9].

**Figure 1:**
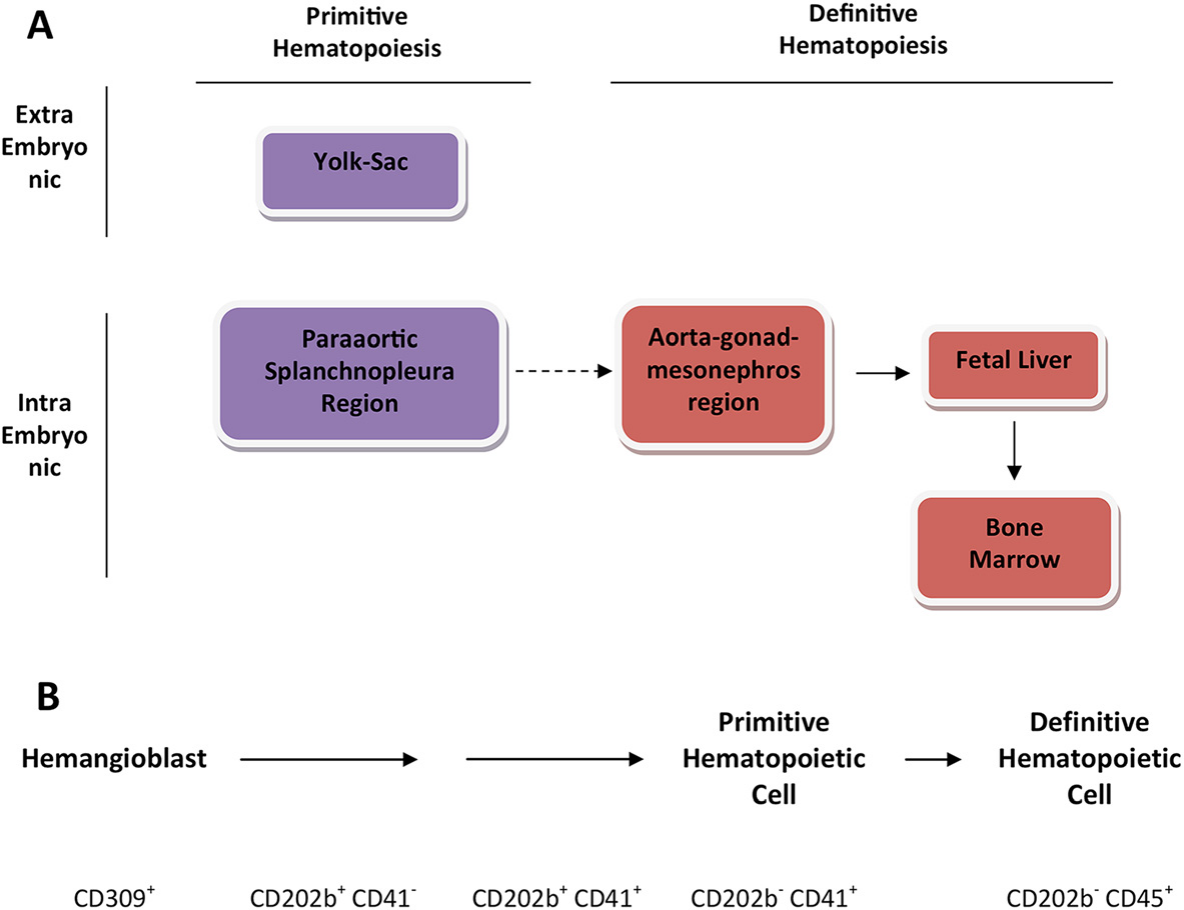
**Primitive and definitive hematopoietic development. (A)** Distinct anatomical regions of primitive and definitive hematopoietic development in both the mouse and human. **(B)** Subset of cell surface markers used in the characterization of this developmental process. CD309, vascular endothelial growth factor receptor 2; CD202b, Tie2 or angiopoietin receptor 2; CD41, integrin 2 alpha; CD45, leukocyte common antigen.

Further studies in mice by Cumano and colleagues sought to address the issue of an anatomical origin of the long-term (LT) reconstituting hematopoietic stem cell (HSC) found in adult animals [10]. Early in murine embryonic development, hematopoietic progenitors can be found in the para-aortic splanchnopleura region on embryonic day 7 (as depicted in Figure 1A), but these progenitors are not LT reconstituting although they do possess lymphoid potential [11-13]. These more advanced hematopoietic progenitors isolated from the aorta–gonad–mesonephros (AGM) region at embryonic day 9.5 to 10 can give rise to lymphoid lineages and are LT reconstituting definitive HSCs, but since this time point is after the establishment of the circulatory system in the embryo their source was difficult to determine [13]. Cumano and colleagues’ approach was to culture explants containing hematopoietic progenitors isolated from the yolk sac and the embryo proper before the onset of circulation, and to determine their LT reconstitution potential [10]. Their results indicated that yolk sac progenitors before the onset of circulation did not have LT reconstitution potential while progenitors isolated from the AGM did, which indicates that primitive and definitive hematopoiesis may occur at distinct anatomical locations [10]. Overall, they demonstrated that the definitive HSC differs from the primitive HSC by lymphoid potential as well as in their ability to achieve LT reconstitution following transfer into adult recipients. More recently, definitive hematopoietic development has also been suggested to possibly occur during fetal ontogeny in subsets of endothelial cells in the heart [14].

## Human hematopoietic development *in vivo*

The study of *in vivo* hematopoietic development in humans is less understood relative to the mouse model due to limitations in experimental approaches. However, evidence resulting from studies in the human embryo has indicated that definitive HSCs emerge in the embryo proper from aortic endothelium similar to observations made in the mouse [15,16]. Morphological studies also observed that HSC clusters can be found within the human aorta, and that these cells express CD34 (sialomucin, a marker to enrich for early hematopoietic progenitors) and CD45 (leukocyte common antigen), markers associated with the HSC fate [15]. Further similarities with the mouse model include the site of initial HSC formation within the aorta and subsequent hepatic colonization [15,17,18], and that definitive hematopoietic cells are derived from within the embryo and not the yolk sac [15,19].

## *In vitro* hematopoietic development

Given the observations made in animal models, several embryonic stem cell (ESC)-based *in vitro* developmental models have been established in the last 20 years. The two arguably most widely used models are the embryoid body system developed by the Keller group [20] and the OP9 co-culture system developed by the Honjo and Nakano groups [21,22].

Embryoid bodies are generated by aggregating ESCs, and cells expressing vascular endothelial growth factor receptor 2 (CD309) are thus induced to differentiate [20]. By isolating CD309-expressing cells and forming an aggregate, a hemangioblast is produced that can give rise to multipotent hematopoietic cells, which can give rise to erythroid, myeloid, and lymphoid lineages but are not capable of LT reconstitution *in vivo* [20,23]. On the other hand, by culturing ESCs with OP9 stromal cells, derived from the *op/op* mice that lack the colony-stimulating factor 1 gene, it is also possible to partially recapitulate hematopoietic development [21]. The products of this developmental model are also multipotent hematopoietic cells capable of erythroid, myeloid, and lymphoid development but not capable of achieving LT reconstitution [21,23]. *In vitro* ESC hematopoietic developmental models therefore clearly lacked fundamental components that could support the development of definitive HSCs in culture, which prompted further research into the signaling pathways that form the basis of this developmental program.

The concept of supporting bone-marrow hematopoietic development with stromal cells is not a new one. Prior to the OP9 stromal co-cultures becoming mainstream, earlier predecessors such as the stromal cultures utilizing mouse kidney cells and cells derived from whole embryo tissue were also noted for their capacity to support myelopoiesis [24]. The importance of supporting stromal cells was demonstrated through use in the eventual identification and isolation of several key cytokines involved in hematopoiesis. Other stromal culturing methods using primary mouse bone-marrow stromal cells coupled to media containing bovine or horse serum, as well as analysis of the impact of steroids in culturing conditions, were also instrumental in optimizing contemporary OP9 stromal culturing conditions [25,26].

## Assays used to study functional outcomes of hematopoietic development

Many methods have been developed to assay the process of hematopoietic development, both *in vitro* and *in vivo*. While there are many protocols for assaying a myriad of molecular and functional outcomes, all of these methods can be categorized into two groups: functional assays and phenotypic/morphological assays.

Phenotypic and morphological assays focus on the characterization of this developmental process by measuring distinct features that appear at specific time points. The oldest technique within this category is microscopy. Reported over 100 years ago and still relevant today, hematopoietic cells are distinguished from the stromal environment by their nonadherent cell morphology [1,27]. Time-lapse microscopy in conjunction with advanced techniques of fluorescent labels has repeatedly demonstrated this process occurring with a marked shifting of cell morphology, from adherent cell to nonadherent cell [7-9]. However, morphological observations have their limitations. The inability to efficiently isolate specific cells from a complex mixture based on appearance poses challenges to define developmental intermediates undergoing hematopoiesis. This shortcoming is addressed with the use of fluorescence-activated cell sorting, which characterizes and isolates cell populations based on their protein expression. Applying this technique to ESC into HSC development, several surrogate markers have been identified to be strongly associated with primitive hematopoietic progenitors and subsequent developmental intermediates. The most well known of these markers is Flk1 (vascular endothelial growth factor receptor 2), found on hemangioblasts that are multipotent progenitors of hematopoietic and endothelial cell lineages [20]. Other cell surface proteins used in the identification of HSCs include the Lineage^−^Sca1^+^cKit^+^ definition (Sca1, lymphocyte antigen 6A/E; cKit, or CD117 stem cell factor receptor) [28]. Some cell surface markers used in this characterization process are outlined in Figure 1B. Finally, because the hematopoietic developmental process is highly regulated and complex, the number of regulatory factors involved is also vast. The challenge of mapping out the regulatory network in an effort to characterize this process by means of distinct signal transduction and transcription regulation pathways is met with experimental techniques commonly found in molecular biology. From the simple assays such as polymerase chain reaction and western analysis to complex and high-throughput gene arrays and chromatin immunoprecipitation sequencing experiments, transcription factors such as runt related protein 1 (Runx1, or acute myeloid leukemia 1) and lim domain binding 1 (Ldb1) have been identified to play crucial roles [29-31].

However, characterization of distinct features that appear during this process – whether this is a protein or gene being expressed or a cell shifting into a specific shape – does not enable functional outcomes to be determined. The definition of a hematopoietic progenitor is one based on function and not on morphology or phenotype. A host of functional assays have therefore been developed and used in conjunction with the methods mentioned above. The gold standard of all functional assays is of course *in vivo* reconstitution. Successful serial reconstitution of immune-deficient animals is the hallmark of a definitive HSC [28]. Short of *in vivo* models, *in vitro* assays such as methyl cellulose and Matrigel have been used to demonstrate hematopoietic and endothelial potential at a clonal level.

While there are many methods available for the functional characterization of cells and populations, the difficulty of determining the pluripotency of a single cell is always present. This challenge can be met directly by assessing the pluripotency and developmental potential of single cells through the analysis of paired daughter cells [32]. This method is perhaps the most direct to functionally assess the developmental potential of progenitors, at the single-cell level. While the method is cumbersome and whether it presents a physiological environment for assessing developmental outcomes is debated, this analysis nonetheless permitted the discovery of key cytokines that can influence lineage fate choices in hematopoietic progenitors.

## Molecular mechanisms of hematopoietic development

At the core of the molecular mechanisms of this highly complex and regulated process is Runx1 (or acute myeloid leukemia 1 protein) [33]. Okuda and colleagues generated a Runx1 knockout mouse and discovered that this genetic perturbation is embryonically lethal [33]. More interestingly, when the mouse was examined closely there were signs of primitive hematopoiesis, but not of definitive hematopoiesis [33]. Runx1 was thus established as a key transcription factor in the definitive hematopoietic development program.

**Figure 2:**
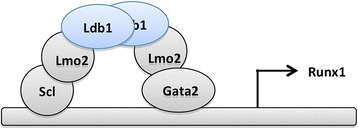
**Transcriptional regulators in the definitive hematopoietic development program.** Lim-domain-binding 1 (Ldb1) stabilizes a complex of transcription factors involving Lim-motif 2 (Lmo2), stem cell leukemia (Scl), and GATA box binding protein 2 (GATA2) [29]. During hematopoietic differentiation, this complex induces the expression of runt related protein 1 (Runx1), which is required for definitive hematopoiesis, as reported previously [27]. Surface marker analysis revealed that generation of CD41^+^ cells is impaired in the absence of Ldb1.

Other transcriptional regulators that act upstream of Runx1 include the Ldb1–Lim motif 2–stem cell leukemia protein–GATA box binding protein 2 protein complex that drives the expression of Runx1 [29,34] (Figure 2). Deficiency in Ldb1 abrogates the expression of Runx1 [29]. Absence of stem cell leukemia leads to a redirection of the hematopoietic developmental program towards the cardiac smooth muscle lineage [30,31]. Finally, GATA box binding protein 2 loss of function also results in embryonic lethality [35]. Downstream of Runx1 are transcription factors that play distinct roles in erythroid, myeloid, and lymphoid developmental programs such as GATA box binding protein 1, PU box binding protein 1 (SPI1), and Runx1 itself, respectively [36-38].

Given that the main components of hematopoietic development depend heavily on multifactorial transcriptional regulating protein complexes that can perform multiple functions depending on the context of their interactions, it is less likely that the hematopoietic developmental program is governed in a linear network connected by distinct signal transduction proteins or protein complexes where defined outcomes arise from a narrow set of inputs. Rather, this process is probably regulated in a probabilistic manner similar to a quantum computer architecture, where a defined input can lead to multiple outputs at varying probabilities based on the context of those inputs. The inputs in this case would be a combination of cell–cell interactions as well as cytokine signals, and the output would be cell fate choices and the associated functional properties. Supporting this notion are studies of de-differentiation and the findings that indicate multiple unstable, dynamically regulated, intermediate states during somatic to embryonic de-differentiation [39]. If there was such a process where environmental cues are kept under full control and development follows a linear path, there would be no stochastic outcomes observed. However, the observations of stochastic developmental results indicate a much more elegant, probabilistic regulatory network that requires far fewer nodes of control and regulation.

## Signaling pathways involved in hematopoietic development

**Figure 3:**
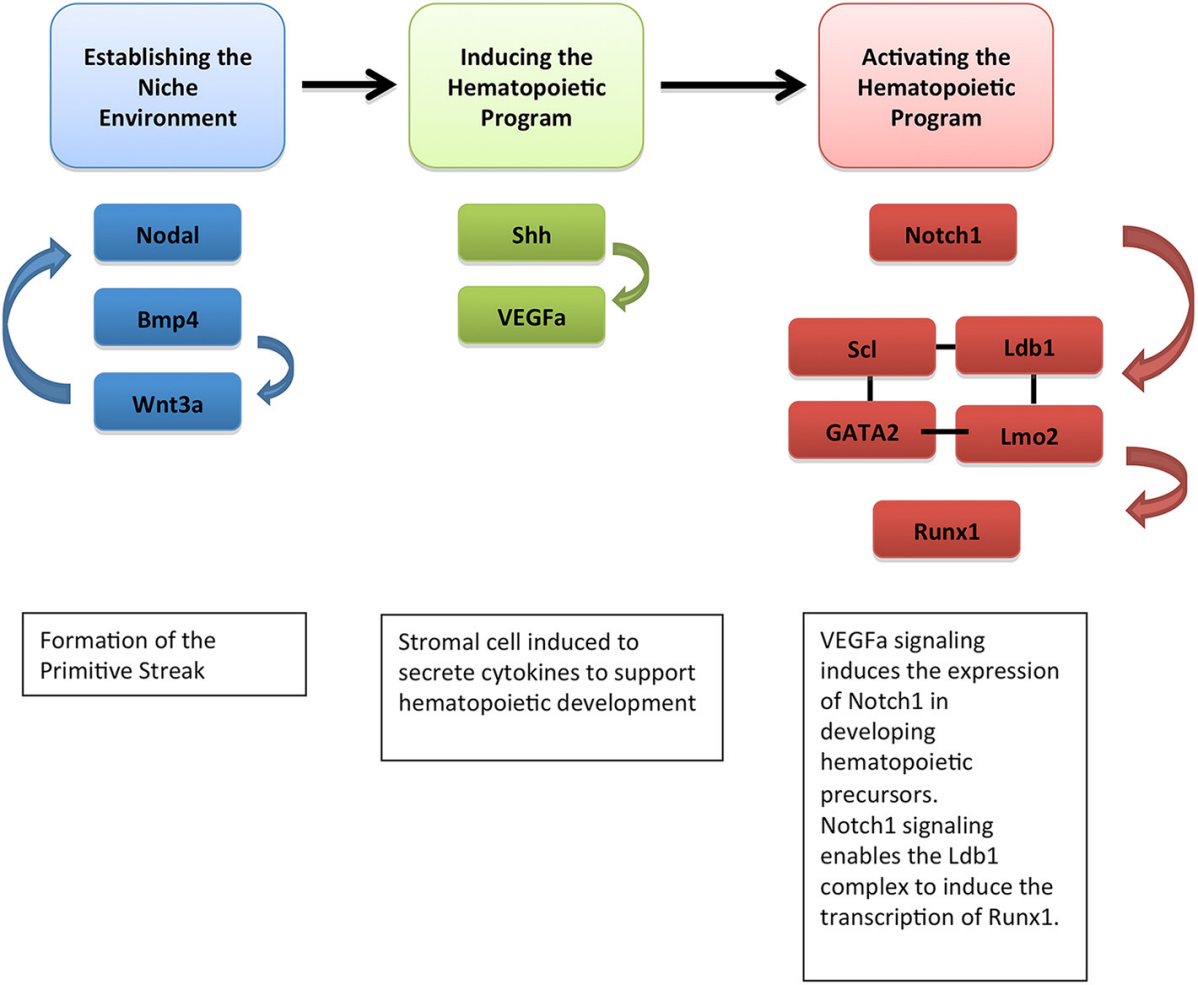
**Signaling factors involved in mammalian embryonic hematopoietic development.** Initially, to form the stromal environment supportive of hematopoietic development, Nodal/bone morphogenic protein 4 (bmp4)/Wnt3a is required for patterning of the primitive streak. Once the stromal environment is established, sonic hedgehog (Shh) secreted by the notochord induces the production of vascular endothelial growth factor alpha (VEGFa) by stromal cells, which signal to activate the transcription of Notch1 in hematopoietic precursors. Notch1 signaling then induces the transcription of members of the Lim-domain-binding 1 (Ldb1) complex, which in turn induces the transcription of runt related protein 1 (Runx1). GATA2, GATA box binding protein 2; Lmo2, Lim-motif 2; Scl, stem cell leukemia; Wnt3a, wingless-type MMTV integration site family, member 3A.

As a complex and highly regulated process, there are multiple signaling molecules – both intrinsic as well as extrinsic – that contribute to the development of an HSC. The best known of these signaling molecules is Notch. Disruption of Notch signaling during embryonic development leads to vascular and cardiac abnormalities, as it is required for the endothelial to mesenchymal differentiation that forms the stromal niche [40-42]. The formation of this stromal niche via Notch1 signaling is required for the embryonic development of HSCs, as well as for HSC specification by inducing the expression of Runx1 possibly through the Ldb1 complex [43,44]. Upstream of Notch1 is signaling via vascular endothelial growth factor alpha by precursors of HSCs, and prior to this are signaling events mediated by sonic hedgehog, which are received by stromal cells and induce the production of vascular endothelial growth factor alpha [44]. Other factors that contribute either to the development or specification of the HSC lineage or to the development of the stromal niche that supports this process include the Nodal/bone morphogenic protein 4/Wnt3a pathways and signaling via fibroblast growth factor [44]. Figure 3 illustrates this process and the potential players involved in early hematopoietic development.

## Hematopoietic stem cell maintenance

**Figure 4:**
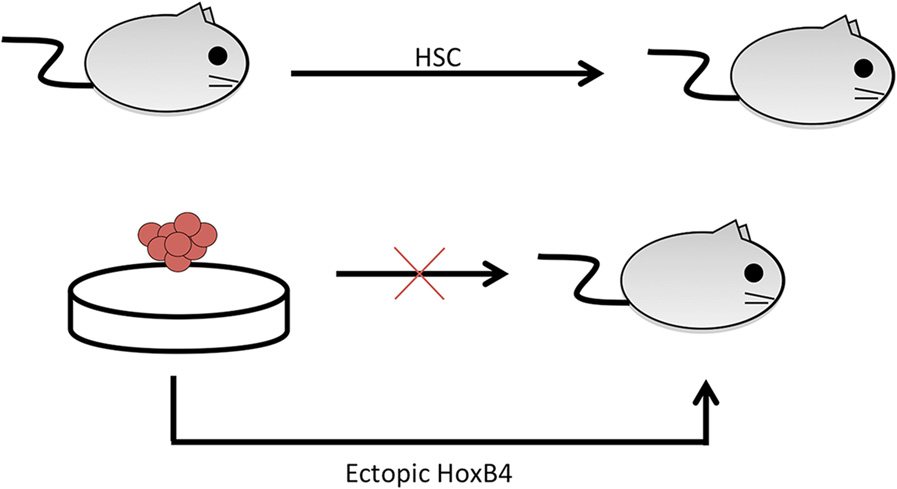
**Hematopoietic stem cell maintenance.** Purified long-term hematopoietic stem cells (HSCs) from adult animals can reconstitute immune-deficient hosts *in vivo*. However, hematopoietic cells generated *in vitro* cannot achieve the same functional outcome. The self-renewal capacity can be induced with the expression of homeobox B4 (HoxB4) ectopically in developing embryonic stem cells, indicating that this potential is present although the trigger is unknown.

As mentioned above, the *in vitro* hematopoietic developmental models designed with ESCs can only achieve a partial recapitulation of the hematopoietic process observed *in vivo*. The key deficiency with *in vitro* models is the lack of LT reconstitution potential in the multipotent hematopoietic cells that are produced [23]. This has been an area of intense research, and the Daley group recently showed that this is possible by activating signaling programs downstream of homeobox protein B4 (HoxB4) [45]. By ectopically expressing HoxB4 in ESCs via retroviral vectors, the Daley group successfully demonstrated that the resulting hematopoietic cells generated *in vitro* could achieve LT reconstitution, as well as maintaining their multipotent property [45] (shown in Figure 4). This observation has prompted research into the role of HoxB4 in hematopoietic development and HSC self-renewal [46-49].

Given the clinical potential for HOXB4 in conditioning HSCs *ex vivo* prior to transplantation, more attention has also been given to the factors that can directly control the expression of HOXB4. One such recent report has implicated GATA box binding protein 2 as a direct upstream regulator of HOXB4 in CD34^+^ cells [50].

## Current challenges and controversies

Despite the achievement of the above-mentioned milestones, much still remains uncertain regarding the origin of the hematopoietic system. Initially morphology and subsequently fluorescence microscopy using tagged proteins, such as CD309 and Runx1, indicated that hematopoietic development occurs in close relationship temporally and spatially with endothelial cell development [1,7-9]. A current issue of contention in this field of research is in the connection between endothelial and hematopoietic development. Hematopoietic cells have been hypothesized to arise from a bipotent progenitor that can give rise to endothelial cells as well as hematopoietic cells, termed a hemangioblast [1,51]. However, there is also a school of thought that hematopoietic cells arise from a unique subset of endothelial cells, called the hemogenic endothelium [51-53]. Evidence from Huber and colleagues and Vogeli and colleagues indicated that a single cell can give rise to endothelial and hematopoietic lineage progeny, and these results served to support the hemangioblast hypothesis [27,54,55]. But there are caveats in this approach, such as the misidentification of progeny that express surface markers commonly associated with mature endothelial cells, and the possibility that pre-hematopoietic precursors may express the same surface proteins cannot be ruled out. On the other hand, evidence for the existence of a hemogenic endothelial cell can be demonstrated by the *in vivo* developmental morphology where a nonadherent hematopoietic cell can be seen budding off from adherent cells in a possibly unique type of cell division [7-9]. This approach also presents uncertainties since morphology does not imply function and the definition of an endothelial cell is mostly by its function as a structural component of the vascular system [56].

*In vitro* studies utilizing genetic modifications that result in blocking hematopoietic development performed by Okuda and colleagues, Chen and colleagues, and our group have shown that it is possible to achieve endothelial cell development in the absence of hematopoietic development [33,57,58]. We also noted that the block in hematopoietic development did not seem to allow a redirection towards the endothelial cell lineage even when the genetic modification occurs upstream of Runx1 expression [58]. Therefore, it is more probable that endothelial and hematopoietic cell development are regulated under distinct but related programs, and may not occur concurrently as the hemangioblast hypothesis would predict.

Other areas of intense debate are found in ESC-derived hematopoietic developmental models, and generally revolve around the common theme of the inability of ESCs to give rise to *in vivo* reconstituting HSCs. The first issue involves the characterization of this process using cell surface markers. While it is well understood that cell surface markers do not dictate the function of the cell expressing them, although occasionally the function of those markers may allow us to infer function, it is nonetheless very useful to identify distinct cell populations based on a defined set of cell surface markers that are expressed or absent. Successful characterization using this approach allows the process to be studied in similar ways as other developmental pathways are studied, by taking full advantage of powerful techniques such as fluorescence-activated cell sorting to isolate distinct subsets of complex population mixtures to test for function both *in vitro* as well as *in vivo*. Over the last several decades, this has not been achieved in a systematic and thorough manner. Initially, CD309 was shown to be expressed by endothelial cells at the site of hematopoietic differentiation *in vivo* [59]. Developing ESCs were then characterized by CD105 (endoglin) expression [60]. In mature HSCs, CD117 and Sca1 expression was used for their identification as Lineage^−^Sca1^+^c-Kit^+^ cells [28]. In all of the abovementioned reports, there may be many markers characterized in total, but in each report only a limited number of markers were analyzed in sparse temporal windows of ESC hematopoietic development. Characterizing the *in vitro* ESC hematopoietic developmental process using all of the abovementioned markers and comparing the results with corresponding *in vivo* subsets would be insightful.

When discussing controversies regarding *in vitro* ESC hematopoietic development, we must also consider the questions posed by the findings using ectopic expression of HoxB4. While the functional outcome is clear, as demonstrated by the Daley group, the mechanism is not well understood [45]. Attempts at dissecting the transcriptional regulatory programs involved following the ectopic expression of HoxB4 in hematopoietic cells derived from ESCs *in vitro* were performed by Oshima and colleagues [49]. By studying the changes in gene expression in these cells, the authors found it possible to elucidate the activated pathways that are activated as a result; however, since HoxB4 was ectopically expressed in ESCs before the onset of differentiation, it is not clear whether the changes in gene expression are the result of HoxB4 expression or a combination of cross-talk between HoxB4-induced programs and feedbacks from the developmental environment. Furthermore, Lee and colleagues also noted that ectopic expression of HOXB4 in human ESCs did not yield the same effect as was observed in murine ESCs [61]. Finding the gap between these two systems will be critical in the translation of results from studies in murine ESC hematopoietic development toward the human system, eventually leading to clinical applications.

## Next steps

These abovementioned unanswered questions all have the same aim, which is the successful generation of a LT reconstituting HSC from an ESC *in vitro*. Although there have been many significant discoveries made on this topic, the next steps in answering these questions will most probably take place in the field of high-throughput genetic assays, followed by functional analysis. In the last decade, we have witnessed an explosion in high-throughput technology that allows us to practically assay gene expression across genomes and test the effects of cytokines, drugs, and chemicals across thousands of biologically active samples. Using these techniques it is possible to design experiments that generate large amounts of data output, which could be analyzed for patterns that may allow inference to function with effective analysis algorithms. High-throughput techniques such as single-cell RNA expression analysis performed by the Gottens group have successfully predicted novel signaling cascades [62]. The number of publications that contain chromatin-immunoprecipitation sequencing, another high-throughput method of mapping the transcriptome of specific populations of cells, as a keyword in 2012 was 788, which is 21 times higher than those of 2008 [63].

Complementing the studies designed to elucidate the transcriptional regulatory networks involved in embryonic hematopoietic development, there is also significant interest in dissecting the epigenetic changes that occur during this process, as well as the comparisons of epigenetic profiles between *in vitro* and *in vivo* derived HSCs. For example, the utilization of Runx1 promoters differs between primitive and definitive hematopoiesis *in vivo* [64]. This change is also accompanied by increased demethylation at the distal promoter, which is more prevalently utilized by definitive HSCs [64]. Findings such as these prompt further investigation into the role of methylation and other epigenetic events in relation to their functional impact upon hematopoiesis both *in vivo* as well as *in vitro*.

Understanding of the cell intrinsic regulatory networks and nodes of control cannot yield a complete picture without consideration being given to cell extrinsic signaling cues. Synchronized with the discoveries made for cell intrinsic signaling pathways and epigenetic changes, there has been considerable recent interest in showing how cytokines can affect the lineage choice of progenitors [65].

## Conclusion

The study of this intricate developmental pathway over the last century has been punctuated by several milestone discoveries: the discovery that this is an evolutionarily conserved process among many species, thus yielding multiple experimental models to explore questions in hematopoiesis; the establishment and fine-tuning of *in vitro* culture models that recapitulate virtually all hematopoietic lineages, allowing questions of hematopoietic potential to be answered; and the advancements in imaging as well as high-throughput methods such as flow cytometry and gene arrays. By studying this system in further detail – genetically, phenotypically, and functionally – its clinical applications are virtually as limitless as the HSCs themselves.

With effective gene therapy, programming subsets of cells such as T cells and B cells to target cancerous or infectious cells and tissues can be applied to potential treatments for congenital, oncological, and transmitted hematological diseases. Translating the method for the generation of HSCs from ESCs to induced pluripotent stem cells would also make possible personalized medical care, where donor matching would no longer be an issue in bone marrow transplantation [66]. One study by the Nakauchi group demonstrated that it is possible to reprogram antigen-specific T cells and renew their functional capacity through induced pluripotent stem cells [67]. As another example that is closer to clinical applications, a biotech in the USA (Calimmune Inc., San Diego, CA, USA) has recently started a clinical trial testing Cal-1-modified hematopoietic stem/progenitor cells for the treatment of HIV [68]. Finally, the characterization of this developmental pathway opens the door to disease modeling where pharmaceutical products can be safely and effectively tested before proceeding with clinical trials in human patients.

## Abbreviations

AGM: Aorta–gonad–mesonephros
ESC: Embryonic stem cell
HoxB4: Homeobox B4
HSC: Hematopoietic stem cell
Ldb1: Lim-domain-binding 1
LT: Long term
Runx1: Runt related protein 1
Wnt3a: Wingless-type MMTV integration site family, member 3A
